# Primary hepatic lymphoma in liver cirrhosis: A rare case report

**DOI:** 10.1016/j.radcr.2021.05.028

**Published:** 2021-06-12

**Authors:** Kaoutar Imrani, Kawtar Znati, Wafae Amouri, Ittimade Nassar, Nabil Moatassim Billah

**Affiliations:** a Radiology Department, Ibn Sina University Hospital, Mohammed V University, Rabat, Morocco; b Department of Anatomo-Pathology, Ibn Sina University Hospital, Mohammed V University, Rabat, Morocco; c Department of Internal Medicine, Ibn Sina University Hospital, Mohammed V University, Rabat, Morocco

**Keywords:** Hepatic lymphoma, Cirrhosis, Imaging

## Abstract

Primary hepatic lymphoma is rare. Clinical and radiological presentations are not specific. The diagnosis is often late. Chronic hepatitis or cirrhosis, especially post-viral C usually precedes primary liver lymphoma. The differential diagnosis arises mainly with other hepatic tumors, such as atypical hypovascular cellular hepatocellular carcinoma when there is liver cirrhosis and with hypovascular hepatic metastases, especially colorectal, stomach and lung metastases. Other differential diagnosis are tuberculosis or sarcoidosis, particularly when there is multiple lesions.

We report the case of a 52-year woman, with a history of hepatitis C infection, presenting liver cirrhosis with multiple hepatic lesions. Radiological aspect was not specific which makes it difficult to distinguish from other hepatic tumors, especially hypovascular liver metastases.

## Introduction

Primary hepatic lymphoma is rare. It is considered when there is no other lymph node or visceral localization. Clinical and radiological presentations are not specific. The diagnosis is often late. Chronic hepatitis or cirrhosis, especially post-viral C usually precedes primary liver lymphoma [1,2].

We report the case of a 52-year woman, with a history of hepatitis C infection, presenting multiple hepatic lesions.

## Case report

52-year-old woman, with a history of hepatitis C infection, was presented for abdominal pain evolving for 4 months in a context of fever and weight loss. The clinical examination found a fever (38.6°C) with splenomegalia, without lymphadenopathy or other associated signs.

**Fig. 1:**
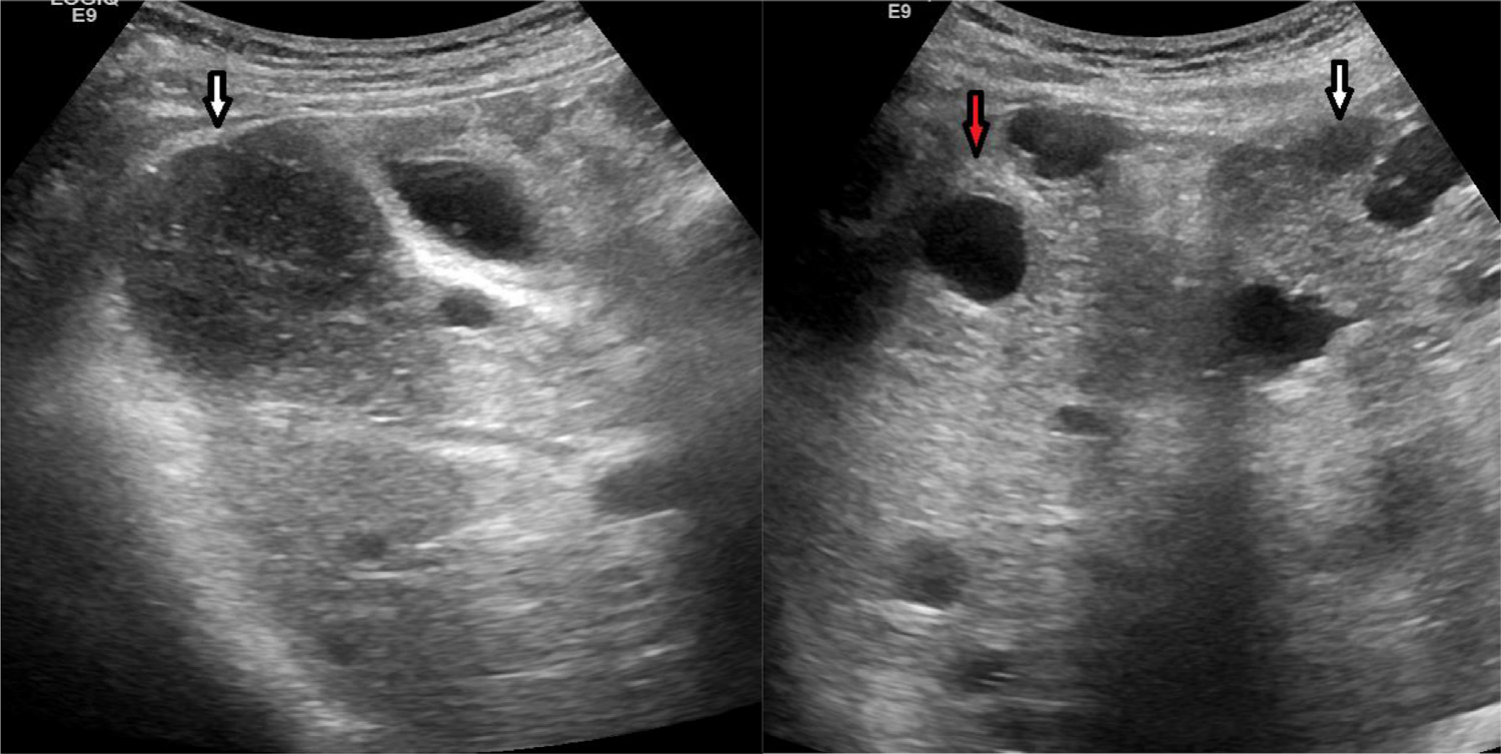
hepatic ultrasound MRI showing multiple cystic lesions (red arrow) associated to hypoechoic nodular lesions (white arrow). (Color version of figure is available online)

Abdominal ultrasound showed a dysmorphic liver with multiple cystic lesions associated to hypoechoic nodular lesions (fig. 1).

**Fig. 2:**
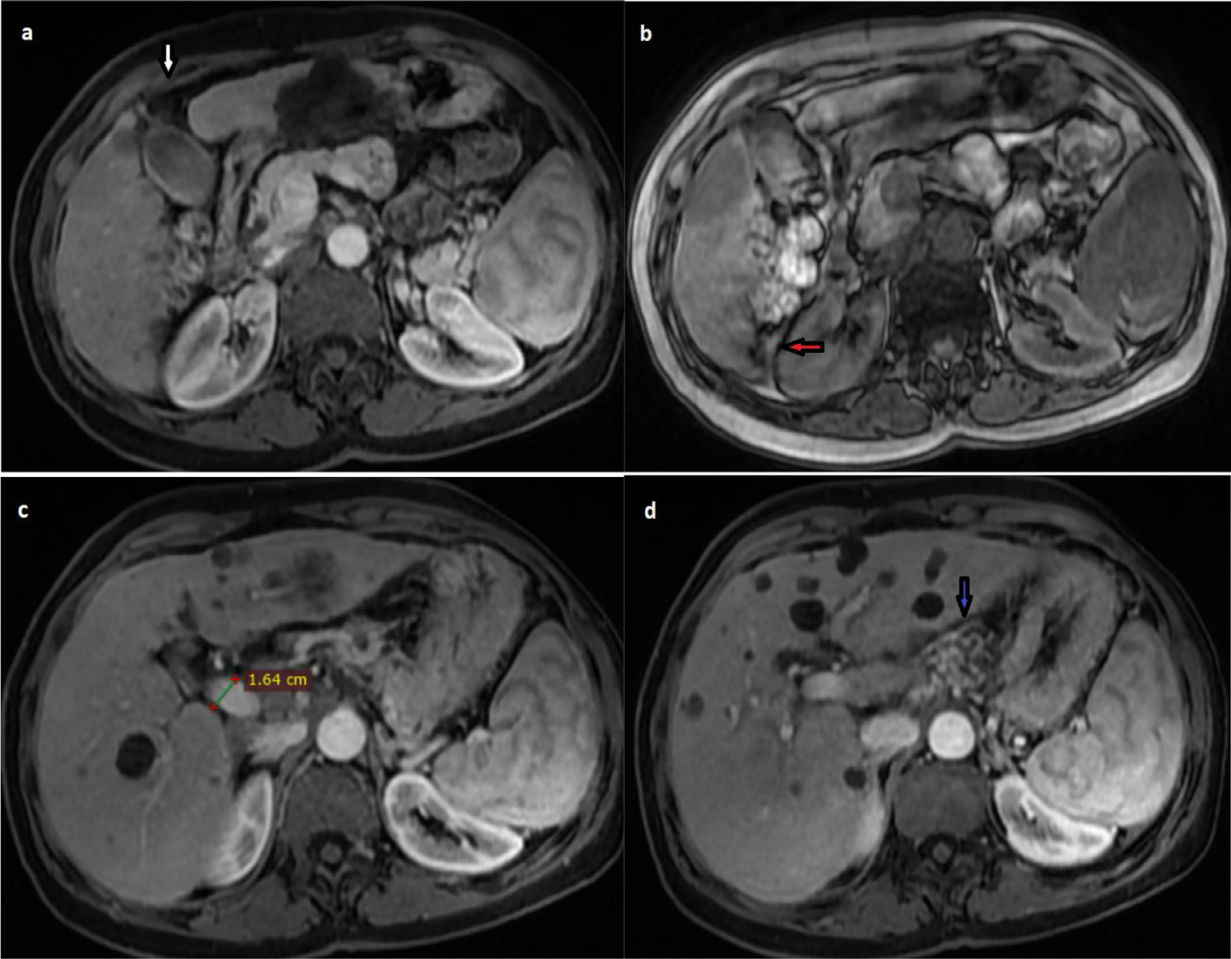
hepatic MRI on T1 post contrast WS (A, C, D) and OP WS (B) showing a cirrhotic liver with hypertrophy of the caudal lobe (note the right liver notch sign [red arrow), and atrophy of segment IV (white arrow), with portal hypertension (dilatation of the portal trunk and portosystemic collaterals [blue arrow]). (Color version of figure is available online)

**Fig. 3:**
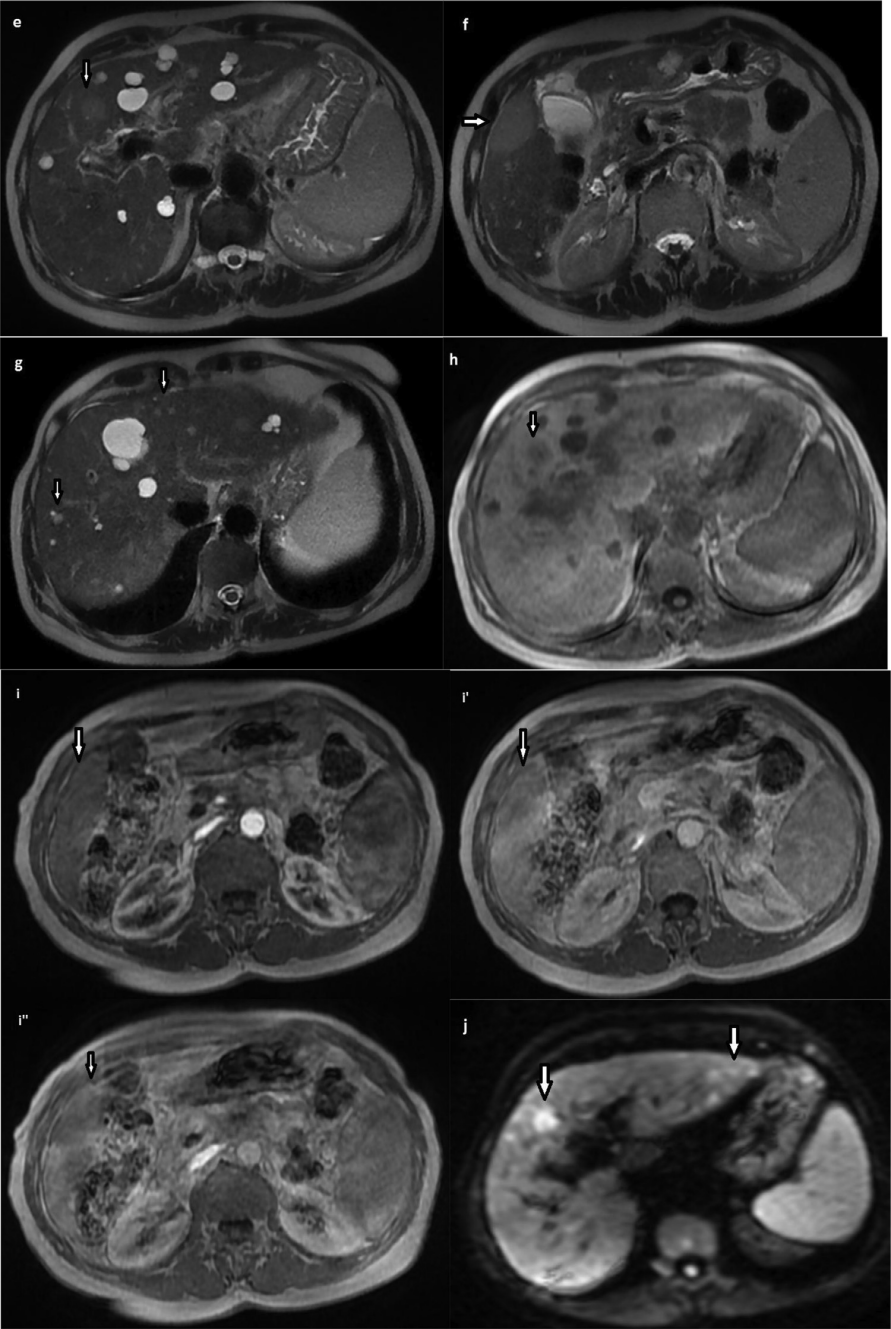
liver MRI on T2-WS (E, F, G), precontrast T1 WS (H), post contrast T1 WS (i:arterial phase, i’:portal phase, i’’:late phase), (I) and diffusion (DWI b = 800 sec/mm2) (J) showing nodular lesions nodular lesions isointense on T1 weighted sequences (WS), with intermediate signal on T2 WS, with late gadolinium enhancement on the periphery, hyperintensity on DWI and hypointensity on apparent diffusion coefficient map, suggesting restricted diffusion.

**Fig. 4:**
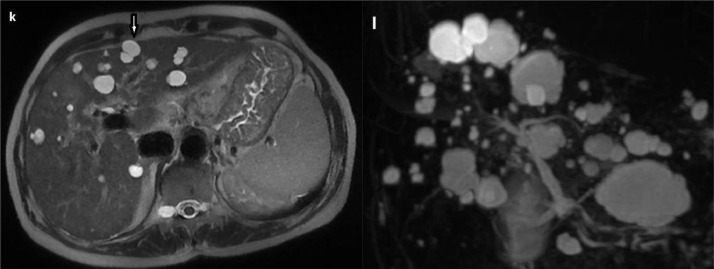
Liver MRI on T2 WS (K) and 3 D MRCP (L)showing multiple hepatic cysts of variable size hyperintense on T2 without communication with the biliary ducts

A liver MRI was performed showing a dysmorphic liver and portal hypertension (fig. 2), with multiple nodular lesions isointense on T1 weighted sequences (WS), with intermediate signal on T2 WS, little enhanced on the periphery after gadolinium injection, with restricted diffusion (fig. 3). It was associated with multiple hepatic cysts hypointense onT1 WS, hyperintense onT2 WS, not enhanced after injection of gadolinium of variable size without communication with the biliary ducts (fig. 4).

There was no lymphadenopathy or splenomegaly.

The aspect of the nodular lesions evoked either atypical hypovascular hepatocellular carcinoma, hepatic metastasis or primary hepatic lymphoma.

Laboratory datas showed normal ranges of blood count cells. There was hepatic cytolysis (alanine aminotransaminase = 300 UI/l, aspartate aminotransferase = 150 UI/L) with normal values of alpha-feto protein (5 ng/ml) and carcinoembryonic antigen (1.5 µg/l). Lactate dehydrogenase was high (400 U/L).

Given the biological and radiological datas, the most likely diagnosis was hepatic lymphoma without being able to eliminate the possibility of hypovascular hepatic metastases.

The thoracic and abdomino pelvic CT scan showed no associated lesions.

**Fig. 5:**
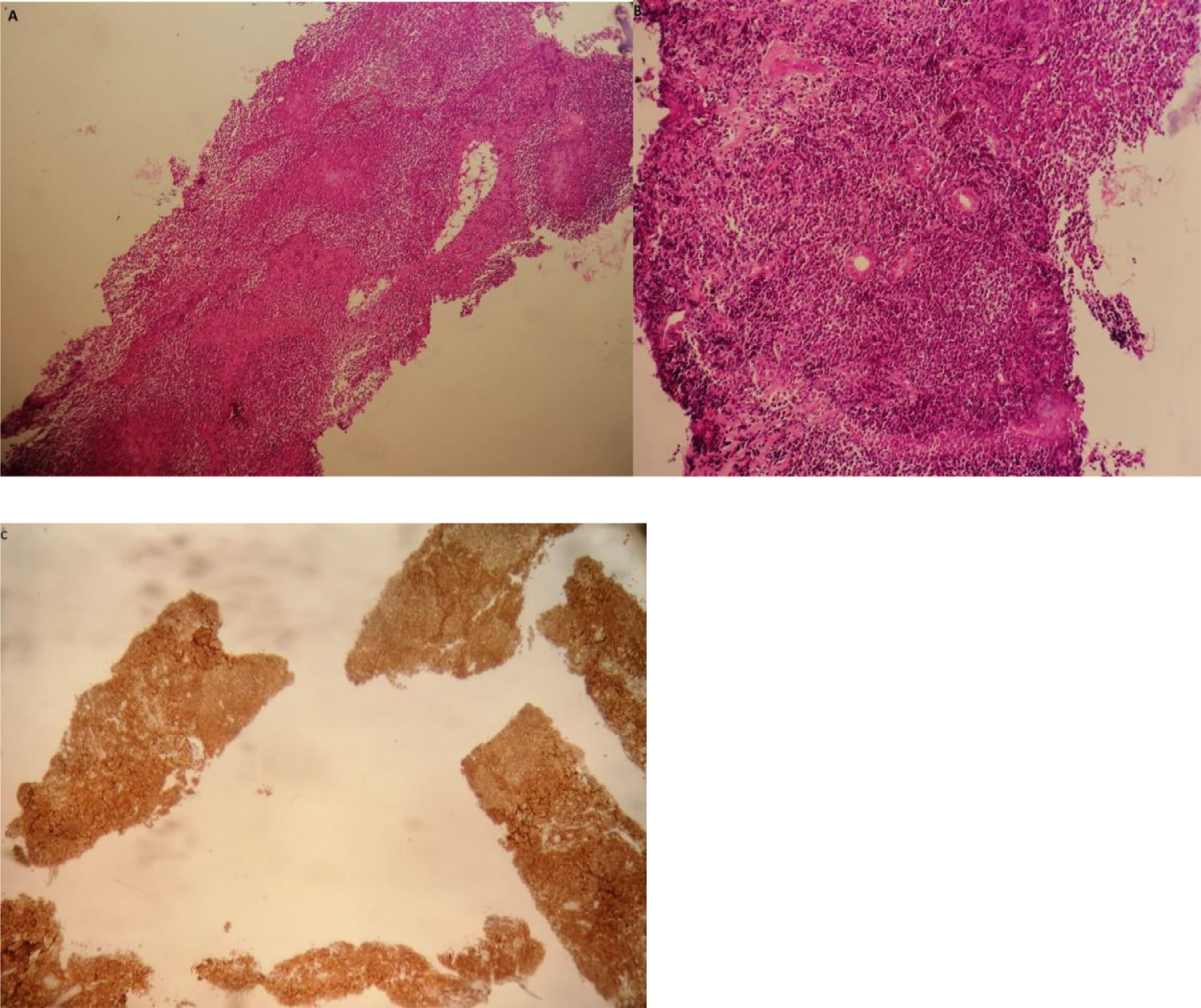
Primary hepatic B lymphoma: (A) histological appearance of the nodule with low magnification showing a dense diffuse proliferation of lymphoid appearance; (B) with higher magnification, the cells evoke centroblasts with oval or multilobed nuclei; (C) tumour cells are positive for CD20, which proves to B cells lineage.

A biopsy of the liver nodular lesions was performed using an 18 Gauge menghini needle. Anatomopathological examination was in favor of B-cell type primary liver lymphoma (fig. 5).

The patient was referred to oncology department for chemotherapy with a good clinical evolution.

## Discussion

Hepatic lymphomas are ususally secondary to systemic impairment. Primary hepatic lymphoma is very rare. It accounts for 0.4% extranodal non-Hodgkin lymphomas and only 0.016% of all non-Hodgkin lymphoma.

Primary hepatic non-Hodgkin lymphomas are defined by the absence of splenomegaly or lymphadenopathy on clinical examination, the absence of lymphoma cells in the blood cell account, a normal chest and abdominopelvic CT scan, with negative bone marrow biopsy and lumbar [2,3]

Chronic hepatitis or cirrhosis, especially post-viral C usually precedes primary liver lymphoma as in our case.

Clinical features are not specific, dominated by alteration of the general state with sometimes hepatic colic or palpable hepatomegaly [4].

Biological assessment may show cytolysis or cholestasis. Lactate dehydrogenase is often high [5].

Imaging is not specific to the diagnosis of hepatic lymphoma which can take three appearances: a solitary nodule, multiple nodules or diffuse hepatic infiltration. According to literature, solitary lesion is the most frequent form followed by multi nodular form. The infiltrative form is not common and is it associated with worse prognosis [1,4,6].

Ultrasound shows hypoechoic lesions compared to hepatic parenchyma sometimes anechogenic.

Abdominal CT shows hypo-dense lesions, not enhanced or showing patchy contrast enhancement [7,8].

On MRI, lesions of primary non-Hodgkin's lymphoma vary in appearance depending on the degree of inflammatory response. They can be iso-intense on T1 WS, hypo, iso or hyper-intense on T2 WS. Contrast enhancement is low or absent. The most frequent appearance is a lesion isointense on T1 WS, hyperintense on T2 WS with peripheral enhancement [1,3,9].

Positron Emission Tomography shows FDG fixation of the liver lesions [7,10].

The differential diagnosis arises mainly with other hepatic tumors, such as atypical hypovascular cellular hepatocellular carcinoma when there is liver cirrhosis and with hypovascular hepatic metastases, especially colorectal, stomach and lung metastases. Other differential diagnosis are tuberculosis or sarcoidosis, particularly when there are multiple lesions. However, in tuberculosis, lesions are small and it is often associated with necrotic lymphadenopathy. In Sarcoidosis, lesions are also small and it is usually associated with typical thoracic manifestations [11,12,13].

The diagnosis is confirmed histologically after liver biopsy. Immunohistochemistry is essential to differentiate lymphoma from other malignant tumors. The vast majority of primary lymphomas in the liver are diffuse large cell lymphomas, mainly type B [2,5].

The therapeutic modalities combine surgery, chemotherapy, radiotherapy or a combination of the different processes. Indications depend on tumour volume, liver function and general condition of the patient [10,14].

## Conclusion

Primary liver lymphoma is a rare entity. The radiological aspect is not specific. The differential diagnosis arises essentially with hypovascular hepatic metastases. This is a diagnosis to keep in mind especially when there is liver cirrhosis without the typical radiological aspect of hepatocellular carcinoma.

## Patient consent

Written informed consent for publication was obtained from the patient. The manuscript does not disclose the patient's private information.
